# Systemic Inflammation and Activation of Haemostasis Predict Poor Prognosis and Response to Chemotherapy in Patients with Advanced Lung Cancer

**DOI:** 10.3390/cancers12061619

**Published:** 2020-06-18

**Authors:** Florian Moik, Sabine Zöchbauer-Müller, Florian Posch, Ingrid Pabinger, Cihan Ay

**Affiliations:** 1 Clinical Division of Haematology and Haemostaseology, Department of Medicine I, Comprehensive Cancer Center Vienna, Medical University of Vienna, 1190 Vienna, Austria; florian.moik@meduniwien.ac.at (F.M.); ingrid.pabinger@meduniwien.ac.at (I.P.); 2 Clinical Division of Oncology, Department of Medicine I, Comprehensive Cancer Center Vienna, Medical University of Vienna, 1190 Vienna, Austria; sabine.zoechbauer-mueller@meduniwien.ac.at; 3 Clinical Division of Oncology, Department of Internal Medicine, Comprehensive Cancer Center Graz, Medical University of Graz, 8036 Graz, Austria; florian.posch@medunigraz.at; 4 I. M. Sechenov First Moscow State Medical University, 119146 Moscow, Russia

**Keywords:** haemostatic biomarker, systemic inflammation, lung cancer, chemotherapy, prognostic model

## Abstract

Systemic inflammation and activation of haemostasis are common in patients with lung cancer. Both conditions support tumour growth and metastasis. Therefore, inflammatory and haemostatic biomarkers might be useful for prediction of survival and therapy response. Patients with unresectable/metastatic lung cancer initiating 1st-line chemotherapy (*n* = 277, 83% non-small cell lung cancer) were followed in a prospective observational cohort study. A comprehensive panel of haemostatic biomarkers (D-dimer, prothrombin fragment 1+2, soluble P-selectin, fibrinogen, coagulation factor VIII, peak thrombin generation), blood count parameters (haemoglobin, leucocytes, thrombocytes) and inflammatory markers (neutrophil-lymphocyte ratio, lymphocyte-monocyte ratio, platelet-lymphocyte ratio, C-reactive protein) were measured at baseline. We assessed the association of biomarkers with mortality, progression-free-survival (PFS) and disease-control-rate (DCR). A biomarker-based prognostic model was derived. Selected inflammatory and haemostatic biomarkers were strong and independent predictors of mortality and therapy response. The strongest predictors (D-dimer, LMR, CRP) were incorporated in a unified biomarker-based prognostic model (1-year overall-survival (OS) by risk-quartiles: 79%, 69%, 51%, 24%; 2-year-OS: 53%, 36%, 23%, 8%; log-rank *p* < 0.001). The biomarker-based model further predicted shorter PFS and lower DCR. In conclusion, inflammatory and haemostatic biomarkers predict poor prognosis and treatment-response in patients with advanced lung cancer. A biomarker-based prognostic score efficiently predicts mortality and disease progression beyond clinical characteristics.

## 1. Introduction

Lung cancer represents the most frequent malignancy globally, with 11.6% of all new cancer diagnoses in 2018 and is responsible for the majority of cancer-related deaths (18.6%) [1]. Most commonly lung cancer, including small cell lung cancer (SCLC) and non-small-cell lung cancer (NSCLC), is diagnosed at an advanced stage (i.e., locally advanced or distant metastatic disease) [2]. Therefore, systemic antineoplastic therapy is the recommended treatment in most patients [3,4]. Despite recent advances in the therapeutic armamentarium, including targeted therapies for specific genetic alterations and the implementation of immune-checkpoint inhibitors in clinical practice, systemic chemotherapy globally remains a fundamental pillar for treatment of patients with advanced lung cancer [3,4]. The ability of clinical characteristics to predict survival and therapy response in patients with advanced lung cancer is limited.

Especially in lung cancer, inflammatory mechanisms play an important role in carcinogenesis and cancer progression [5,6,7]. Biomarkers of systemic inflammation including c-reactive protein (CRP), lymphocyte-monocyte ratio (LMR), neutrophil-lymphocyte ratio (NLR) and lymphocyte-platelet ratio (PLR) as indicators of the cellular-mediated inflammatory response, have been reported to be associated with survival in different subgroups of patients with lung cancer [8,9,10,11,12].

Interestingly, also the haemostatic system has been suggested to support a tumour-promoting microenvironment in lung cancer. For instance, the expression of tissue factor and proteinase-activated receptors in lung cancer have been linked to impaired apoptosis on a cellular level, worse survival and the metastatic process [13,14,15,16,17]. Further, the haemostatic system has been demonstrated to be involved in angiogenesis, tumour growth and metastatic spread in experimental models of lung cancer [18,19]. More recently, elevated biomarkers of haemostasis (plasma fibrinogen and soluble (s)P-selectin) have been associated with risk of lung cancer in a prospective observational study, underpinning this complex interrelation [20]. Haemostatic biomarkers and tests, such as prothrombin time, fibrinogen and D-dimer levels have further been reported to be associated with survival in patients with lung cancer [21,22,23,24].

The aim of this prospective study was to assess the association of a comprehensive panel of markers representing an inflammatory and haemostatic state with prognosis and therapy response in a large cohort of patients with lung cancer initiating chemotherapy. Thereby, we aimed at adding profound clinical evidence to the mechanistic concept of the interrelation between lung cancer and the inflammatory and haemostatic system. By utilising a broad panel of biomarkers, we aimed at improving the ability to predict survival and therapy response beyond clinical variables and thereby contribute to personalized clinical decision making.

Our panel of biomarkers was selected based on previous reports on their prognostic value in patients with cancer. Markers of haemostasis chosen for our present study include D-dimer [23,25,26] and prothrombin fragment 1+2 (F1+2) [25] as indicators of in-vivo haemostatic activity, coagulation factor VIII activity (FVIII) [27] and peak thrombin generation (peak-TG) [28] as indicators of systemic hypercoagulability, soluble sP-selectin [29] as a marker of platelet activation and fibrinogen [30,31]. Inflammatory biomarkers include CRP as a marker of systemic inflammation and NLR, LMR and PLR as indicators of the cellular inflammatory response [11,12,17,32]. Blood count parameters of interest included haemoglobin, leucocytes and thrombocytes as markers for chronic disease, inflammation and immune dysregulation [33,34,35,36]. Further details on our panel of biomarkers are provided in Appendix A.

## 2. Results

### 2.1. Baseline Characteristics and Therapeutic Data of the Study Population

Of the 277 patients included in the present study, 103 were female (37.2%) and the median age of the cohort was 61 years (interquartile range (IQR): [56, 67]). At study inclusion, the median performance index according to the Eastern Co-operative of Oncology Group (ECOG) was 1 (IQR: [1, 2]) (Table 1). Most patients were newly diagnosed with lung cancer at the time of study inclusion (*n* = 218, 78.8%) and the remainder of patients had recurrent disease (*n* = 59, 21.3%). The most frequent lung-cancer subtype was NSCLC in 246 (83.4%) patients, comprising mostly patients with adenocarcinoma (*n* = 165; 59.6%) and squamous-cell carcinoma (*n* = 45; 16.2%). Forty-six (16.6%) patients had SCLC. Eight patients were found to have targetable mutations (2.9%). Most patients presented with distant metastatic disease (*n* = 196; 70.8%), and the remaining 81 (29.2%) patients initiated systemic chemotherapy due to locally advanced disease or unresectable disease. The median number of chemotherapy cycles applied during 1st line chemotherapy was 4 (IQR: [2, 5]). The most frequently used chemotherapeutics were platinum-based agents, mostly in combination with Vinorelbine (*n* = 74; 26.9%), with Gemcitabine (*n* = 52; 18.9%), with Pemetrexed (*n* = 52; 18.9%) or Etoposide (*n* = 49; 17.8%). Six patients (2.2%) received vascular endothelial growth factor (VEGF) targeted therapy, while 10 (3.6%) patients received epidermal-growth-factor receptor tyrosine-kinase inhibitors (EGFR-TKI). Baseline characteristics, tumour specific data and therapy related parameters are summarized in Table 1, including the distribution of all haemostatic and inflammatory parameters included in our panel of biomarkers.

### 2.2. Overall Survival and Therapy Response

Over a median follow-up of 24 months, 197 deaths (71.1% of the study cohort) were observed. Median OS in the overall study cohort was 12.3 months [95% confidence interval (CI): 10.5–14.5], with 6-, 12-, and 24-month survival estimates of 78.3% [95% CI: 73.0–82.7], 52.0% [95% CI: 46.0–57.7] and 27.9% [95% CI: 22.7–33.4], respectively. Median PFS was 5.5 months [95% CI: 4.6–6.4] and the rate of patients who achieved radiological remission or stabilisation of disease during 1st-line chemotherapy (i.e., DCR) was 65.4% (*n* = 174). Clinical parameters that were associated with increased mortality in univariable analysis included stage of disease (stage IV vs. others, hazard ratio (HR): 2.62 [95% CI: 1.83–3.76]), occurrence of VTE (transition HR for death after VTE within a multi-state-model: 2.50 [1.55–4.01]) and higher ECOG performance status (HR for ECOG > 2 vs. ECOG ≤ 2: 1.52 [95% CI: 1.03–2.24], whereas no association with mortality was observed for histological grade (HR 1.12 [95% CI: 0.85–1.46]), age (HR per 10 years increase: 1.13 [95% CI: 0.96–1.33]), sex (HR for male sex: 1.20 [95% CI: 0.90–1.61]) or histological differentiation (NSCLC vs. SCLC, HR: 1.19 [95% CI: 0.81–1.75]).

### 2.3. Association of Biomarkers with Mortality

We evaluated the association of our selected biomarkers with risk of death per doubling of levels on a continuous scale. A multivariable analysis was conducted, adjusting for histology, stage, sex, age, respiratory comorbidity, presence of targetable mutation and occurrence of VTE. Haemostatic biomarkers found to be independent predictors of mortality were D-dimer (HR per doubling of levels: 1.50 [95% CI: 1.29–1.75], sP-selectin (1.42 [1.09–1.83]) and FVIII (1.46 [1.08–1.98]). No significant association with risk of death was observed for levels of F1+2, fibrinogen and peak-TG (Table 2).

Inflammatory biomarkers and blood count parameters found to be independent predictors of mortality were haemoglobin (HR: 0.13 [0.06–0.30]), lymphocyte count (HR: 0.72 [0.57–0.91]), NLR (HR: 1.24 [1.03–1.49]), LMR (HR: 0.60 [0.45–0.80]), PLR (HR: 1.31 [1.08–1.61]) and CRP (HR: 1.45 [1.27–1.65]). No significant association with risk of death was observed for platelet count, leucocytes, neutrophil granulocytes and monocytes. Table 2 displays detailed results from uni- and multivariable analysis of the association between our panel of haemostatic and inflammatory biomarker with mortality.

Adjustment for multiple testing according to Bonferroni and Holm was conducted for analysis of mortality, the primary outcome of the study. In multivariable analysis after adjustment of the alpha level for multiple testing, a significant association with mortality was observed for levels of D-dimer, haemoglobin, LMR and CRP.

### 2.4. Association of Biomarkers with Disease Progression and Disease Control Rate

In multivariable analysis, levels of D-dimer (HR per doubling of levels: 1.34 [1.16–1.53]), F1+2 (HR: 1.22 [1.04–1.44]), haemoglobin (HR: 0.28 [0.14–0.59]), lymphocyte count (HR: 0.76 [0.61–0.93]), NLR (HR: 1.24 [1.05–1.46]), LMR (HR: 0.74 [0.58–0.94]), PLR (HR: 1.24 [1.03–1.48]) and CRP (HR: 1.25 [1.11–1.40]) were associated with increased risk of disease progression. Levels of sP-selectin, fibrinogen, FVIII, peak-TG, platelet and leucocyte count, neutrophils and monocytes were not associated with risk of disease progression per time (Table 2).

Probability to achieve radiological disease control (i.e., DCR) during 1st line of chemotherapy was increased in multivariable analysis by higher levels of haemoglobin (odds ratio (OR) per doubling of levels: 10.94 [2.26–53.1]) and decreased by higher levels of CRP (OR 0.69 [0.53–0.89]). Elevating levels of D-dimer (OR: 0.73 [0.52–1.04]) and F1+2 (OR: 0.71 [0.50–1.02]) were numerically associated with a decreased probability to achieve disease control, however only with borderline statistical significance. No significant association with DCR was observed for levels of sP-selectin, fibrinogen, FVIII, peak-TG, platelet and leucocyte count, neutrophils, lymphocytes, monocytes, NLR, LMR and PLR. Table 2 displays detailed results of the uni- and multivariable analyses of all investigated biomarkers towards PFS and DCR.

### 2.5. Derivation of a Biomarker Based Prognostic Model

From our comprehensive panel of investigated biomarkers, a unified prognostic model was derived. We identified levels of D-dimer, LMR and CRP as optimal variables for inclusion in our model (HRs in the unified final model: D-dimer: 1.35 [95% CI: 1.12–1.62], LMR: 0.63. [95% CI:0.46–0.89], CRP: 1.38 [95% CI: 1.17–1.63]). These parameters were selected from all independently predictive biomarkers for mortality from our primary analyses (Table 2) by backwards elimination in a joint Cox-regression model with a cut-off for omitting variables at α = 0.157, and therefore, represents the best fit prognostic model based on the Akaike information criterion (AIC) [37].

The log-transformed hazard ratio (logHR) from the final model was used to weigh levels of each biomarker according to its individual strength of association with mortality. Individual prediction of mortality could be obtained according to the following Equation (1), including levels of D-dimer in mg/dL, LMR as the ratio of absolute lymphocyte count divided by absolute monocyte count and CRP in mg/dL:

(1) $$1yrmortality=100\times \left(1-0.5862767^{exp\left(linearpredictor\right)}\right)\;\begin{array}{cc} linearpredictor & =0.2967\times \frac{ln\left(ddimer\right)}{ln\left(2\right)}-0.4472\times \frac{ln\left(lmr\right)}{ln\left(2\right)}\\ & +0.3224\times \frac{ln\left(crp\right)}{ln\left(2\right)} \end{array}$$

**Figure 1:**
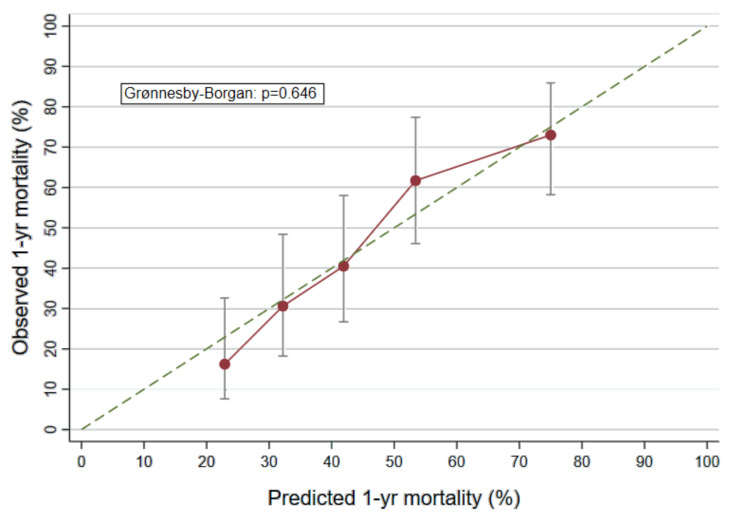
Calibration plot of the biomarker based prognostic model. This graph plots observed against predicted 1-year risk of mortality within quintiles of the linear predictor of our biomarker-based prognostic model. Smaller distance between scatter points from the 45° reference line (dotted) indicate better calibration. Scatter points are displayed with 95% confidence intervals of observed mortality.

Internal validation by bootstrapping was performed and the model achieved an adjusted concordance index (Harrel’s C) of 0.68 [95% CI: 0.64–0.72]. Coefficients of the final model were stable upon bootstrapping. Graphical inspection of the association between predicted and observed mortality and a *p*–value of the Grønnesby–Borgan test of 0.646 indicated good model calibration (Figure 1).

**Figure 2:**
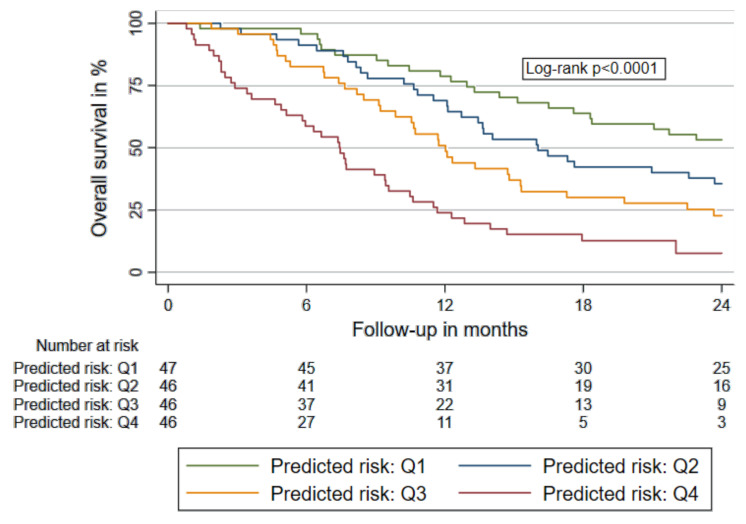
Overall survival according to the biomarker-based prognostic score. The prognostic model comprises weighted levels of D-dimer, LMR and CRP. Patients were stratified in quartiles according to individual predicted risk of mortality. Abbreviations: LMR: lymphocyte-to-monocyte ratio; CRP: c-reactive protein; Q1: first quartile (lowest predicted risk, ≤25th percentile); Q2: second quartile of predicted risk (>25th percentile and ≤50th percentile); Q3: third quartile (>50th percentile and ≤75th percentile); Q4: fourth quartile (>75th percentile).

One-year OS stratified by quartiles of predicted risk (i.e., quartiles of the linear predictor) were 79%, 69%, 51% and 24%, respectively. Two-year OS according to quartiles of the predicted risk were 53%, 36%, 23% and 8%, respectively. Median OS was 7.4 months [95% CI: 4.9–9.4] in patients in the highest-risk quartile of the prognostic model, 12.0 months [9.2–15.3] in those within the third quartile, 16.0 [12.1–23.7] in those within the second quartile and was not reached in patients in the lowest-risk quartile, indicating that >50% in this subgroup were alive after 24 months of follow up. Figure 2 displays survival functions of patients according to our biomarker-based prognostic model.

In order to facilitate interpretation and reproducibility, a simplified point-based score was developed. Patients were assigned +2 points per increase in D-dimer of 1 mg/dL, +3 points per increase in CRP of 1 mg/dL and −5 points per increase in the LMR of 1. The median score was −2 points (IQR: [−11, +5]; range: [−141, +93]). Risk of mortality was elevated for increasing scores (HR per 10 points increase: 1.21 [95% CI: 1.12–1.30], *p* < 0.001).

An independent association of the biomarker-based score with mortality beyond clinical risk factors was observed in multivariable analysis adjusting for histology, stage, sex, age, respiratory comorbidities, presence of targetable mutations and VTE (HR per 10 points increase: 1.18 [95% CI: 1.10–1.28], *p* < 0.001). We further evaluated the prognostic capability of our biomarker score beyond an established risk score based on clinical risk factors in patients with NSCLC, the m-LCPI. Biomarker scores increased by m-LCPI risk categories, with a median score of −16 points (IQR: [−17, −10]) in category 1, −6 points (IQR: [−15, +2]) in category 2, −1 points (IQR: [−9, +7]) in category 3 and 3 points (IQR: [−1, +16]) in category 4. Upon multivariable adjustment, the biomarker score was associated with mortality beyond the m-LCPI risk score (HR for 10 points increase: 1.21 [1.10–1.33], *p* < 0.001). To evaluate the additive prognostic utility beyond this validated clinical model, a comparative analysis of discriminatory indices was conducted. Implementation of the biomarker-based score in the Cox model led to an increase of Harrell´s C from 0.56 [95% CI: 0.52–0.60] to 0.68 [95% CI: 0.63–0.74], indicating a 12% increase in discriminatory performance.

**Figure 3:**
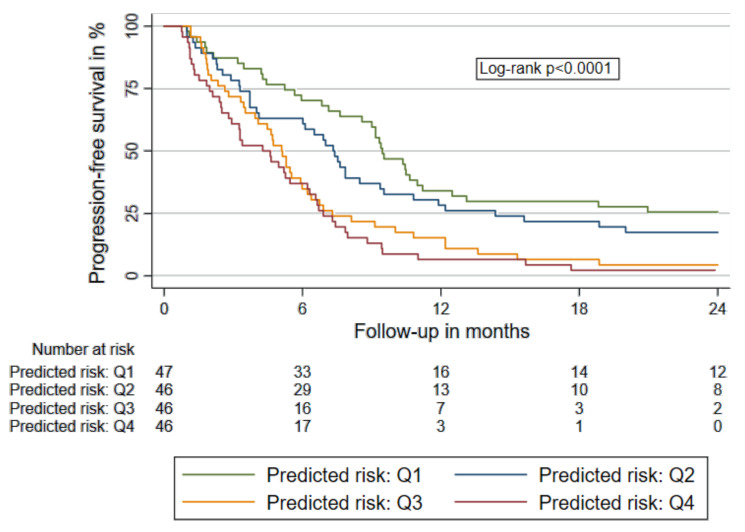
Progression free survival according to the biomarker-based prognostic model. The prognostic model comprises weighted levels of D-dimer, LMR and CRP. Patients were stratified in quartiles according to individual predicted risk of mortality. Abbreviations: LMR: lymphocyte-to-monocyte ratio; CRP: c-reactive protein; first quartile (lowest predicted risk, ≤25th percentile); Q2: second quartile of predicted risk (>25th percentile and ≤50th percentile); Q3: third quartile (>50th percentile and ≤75th percentile); Q4: fourth quartile (>75th percentile).

We further explored the predictive capability of our biomarker-based prognostic model towards response measures to chemotherapy (PFS and DCR). Elevation of predicted risk within our biomarker-based prognostic model was associated with an increased risk of disease progression (HR: 2.02 [95% CI: 1.57–2.62], *p* < 0.001) and a decrease in the probability to achieve disease control during 1st line chemotherapy (OR for DCR: 0.57 [0.34–0.97], *p* = 0.038). In Figure 3 survival functions of PFS according to our biomarker-based prognostic model are displayed.

## 3. Discussion

We investigated a large and comprehensive panel of biomarkers within a prospectively followed cohort of patients with advanced lung cancer initiating systemic chemotherapy. Our results suggest that haemostatic activation and systemic inflammation are related to poor prognosis and unfavourable therapy response profiles. In detail, increased pre-chemotherapeutic levels of D-dimer, sP-selectin, FVIII, haemoglobin, lymphocytes, NLR, LMR, PLR and CRP were associated with decreased survival in patients beyond clinical risk factors. Further, elevated levels of D-dimer, F1+2, haemoglobin, lymphocyte count, NLR, LMR, PLR and CRP were associated with shorter time to progression of disease. Increasing levels of haemoglobin and CRP were significantly associated with lower disease control rates during chemotherapy, whereas haemostatic biomarkers (D-dimer, F1+2) predicted for a lower probability of disease control with borderline significance.

Improved prognostic accuracy is crucial in order to support individualized therapeutic decision-making, in identifying high risk populations for closer monitoring or for selecting patients for clinical trials based on predicted survival times. From our panel of inflammatory and haemostatic parameters, we derived a biomarker-based prognostic model. This model is based on the strongest individual prognostic biomarkers within our panel and comprises weighted levels of D-dimer, LMR and CRP, which represent easily available parameters in clinical practice. Our model was internally validated and independently associated with mortality upon adjustment for key clinical risk factors. Further, our model efficiently predicted survival beyond the m-LCPI, an established risk score based on clinical characteristics in the subgroup of patients with NSCLC [38]. To date, the possibility to predict survival in patients with advanced lung cancer is limited. The m-LCPI was derived in a heterogeneous population of patients with NSCLC and is largely based on tumour stage [38]. As patients with advanced lung cancer who initiate systemic anticancer therapy are homogeneous in respect to several key clinical characteristics including stage of disease, our goal was to improve the prognostic accuracy in this specific subpopulation.

Biomarker based prognostic models including the Glasgow Prognostic Score (GPS) and its modification (mGPS), based on CRP as a marker of systemic inflammation and albumin as an indicator of malnutrition, have been validated in various oncological settings, including lung cancer and patients with advanced NSCLC [39,40,41]. However, the derivation of this score was limited in regard to the number and types of investigated biomarkers and our score adds the strong prognostic capability of haemostatic biomarkers to these inflammation-based models.

In addition to its prognostic capability towards mortality, our biomarker-based model predicted for response to chemotherapy. Thereby, we underline our observation of an association between selected haemostatic and inflammatory biomarkers with PFS and DCR in our single-biomarker analysis. This indicates a potential increase in resistance to chemotherapy in patients with lung cancer and profound systemic inflammation and haemostatic activation.

Our results support the pathophysiological concept of an important interrelation of lung cancer with the haemostatic and inflammatory system. Increased coagulation and inflammation seem to be associated with increased tumour aggressiveness, as characterized by shorter survival times and worse response to systemic anticancer therapy. Results of experimental and translational work suggest an underlying causal relationship by linking tumour-induced inflammation and hypercoagulability to cellular pathways and alterations in the tumour microenvironment that support proliferation, invasion, metastasis and the evasion of apoptotic mechanisms [6,7,13,14,15,18,19]. These findings are strongly supported by our observations that add a valuable clinical perspective to our understanding of the complex interplay of inflammation, haemostasis and lung cancer.

Despite various strengths of our study, including the variety of biomarkers evaluated, prospective follow-up and state-of-the-art model derivation and internal validation, several limitations must be addressed. First, despite internal validation of the biomarker-based prognostic score, external validation of our findings must prelude potential clinical applicability of these results. Secondly, despite prospective follow up for our primary outcome variable of overall survival, therapy response data were gathered in retrospect by inquiring restaging imaging procedures and electronic medical charts. However, as of the completeness of follow-up data and the routine nature of these outcome variables in oncological patients, we have high confidence in the integrity of therapy response data. Thirdly, by evaluating a broad panel of biomarkers, the probability of an inflation of the family-wise error rate due to multiple testing is increased. By adjusting for multiple testing this issue was controlled for in our primary outcome analysis. However, due to the exploratory nature of our analysis towards secondary outcome parameters, results of the association of biomarkers with therapy response must be considered hypothesis-generating. Lastly, the inclusion of specific inflammatory biomarkers such as IL-6 and TNF-α might have added prognostic value to our panel. However, we believe our selection of inflammatory markers is reflective of the tumour-inflammation axis on a systemic level, and we could show that both cellular inflammatory biomarkers like the LMR and plasmatic biomarkers like CRP were effective in predicting survival and therapy response in our cohort. Despite these limitations, our study markedly contributes to a broader understanding of the interrelation of the haemostatic system, the inflammatory system and advanced lung cancer on a clinical scale.

## 4. Materials and Methods

### 4.1. Study Design and Procedures

This study was conducted within the framework of the Vienna Cancer and Thrombosis Study (CATS), a prospective observational single-centre study. All subjects gave their informed consent for inclusion before they participated in the study. The study was conducted in accordance with the Declaration of Helsinki, and the protocol was approved by the Ethics Committee of the Medical University of Vienna (Project identification code: 126/2003, date of approval: 02 September 2003).

Patients with histologically confirmed malignancy, either newly diagnosed or recurrent/progressive disease after complete or partial remission, were eligible for inclusion. Importantly, exclusion criteria included clinically overt infection within 2 weeks, venous or arterial thromboembolic events within 3 months, continuous anticoagulation, surgery or radiotherapy within 2 weeks or chemotherapy within 3 months prior to inclusion in order to prevent confounding of baseline biomarker measurements. Further details on design, in- and exclusion criteria are provided within Appendix B and have been reported previously [25,29].

Upon inclusion, patients provided written informed consent for study participation and underwent a structured interview about comorbidities and medical history. Disease- and therapy-related data were documented by chart review. Prior to initiation of chemotherapy, baseline blood sampling was performed, and biomarker measurement was conducted either the same day or plasma-aliquots were stored at −80 °C until testing was performed in series. Details on methods of biomarker measurement are provided in Appendix C.

Patients were prospectively followed for a maximum of 2 years. The original primary endpoint of CATS is venous thromboembolism (VTE), with death from any cause as a key secondary endpoint. For the present study, mortality, defined as rate of death from any cause and overall survival (OS), defined as time to death were set as main outcomes. Data on date and cause of death were gathered by inquiring the official Austrian Death Registry. Secondary outcome parameters for the present study were progression-free-survival (PFS), defined as time to radiological progression of disease (according to response evaluation criteria in solid tumours, version 1.1 (RECIST 1.1)) [42] and disease-control-rate (DCR), defined as the rate of patients with either complete or partial remission or stable disease according to RECIST 1.1 during 1st line of chemotherapy. Data on therapy response were collected from electronic medical records and original reports of radiological restaging procedures, as interpreted by the treating oncologist.

### 4.2. Derivation of Study Cohort

**Figure 4:**
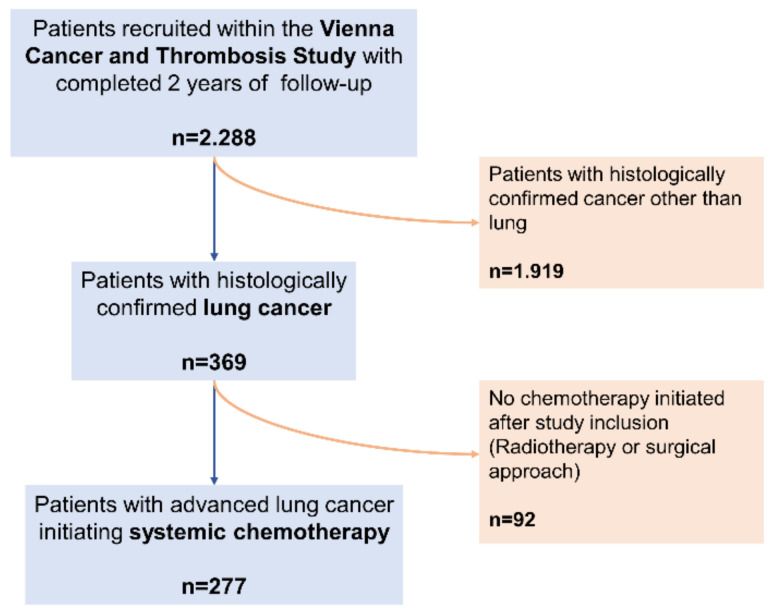
Flow-chart of patients included in the study.

Until July 2019, 2288 patients were included in CATS and completed follow-up, including 369 patients with histologically confirmed lung cancer. Of those, 277 patients who initiated systemic chemotherapy after inclusion due to metastatic (*n* = 196) or locally advanced/unresectable disease (*n* = 81) were included in the present study (Figure 4).

### 4.3. Statistical Analysis

Time-to-event analysis was performed by the method of Kaplan–Meier, using log-rank test for between group differences. Uni- and multivariable modelling of the association of biomarkers per double increase on a continuous scale with study-outcomes was conducted by means of Cox-regression (OS and PFS) and binary logistic regression (DCR). The selection of co-variables for multivariable adjustment was based on previous knowledge on clinical variables that might confound biomarker analysis and include histology (NSCLC or SCLC), stage of disease, sex, age, presence of respiratory comorbidities, presence of targetable mutation, and occurrence of VTE (with VTE as time-dependent co-variable). Mortality analysis was adjusted for multiple testing according to Bonferroni–Holm. No post-hoc test for multiple testing was conducted for analysis of secondary outcomes (PFS; DCR) [43].

A comprehensive panel of selected biomarkers was included in the derivation of a prognostic model. For model derivation, backwards elimination with a cut-off for omitting variables of alpha = 0.157 was conducted to identify the optimal model according to the Akaike information criterion (AIC) within nested hierarchical models [37]. The prognostic model comprises baseline survival functions and biomarker-specific weighting factors, representing the individual strength of association with mortality based on the logarithm of the hazard ratio (HR). Discrimination of the final prognostic model was assessed by the concordance-index Harrell´s C and calibration was assessed graphically by plotting observed against predicted mortality within quintiles of the linear predictor (Figure 2) [44,45,46]. Goodness-of-fit was assessed by the Grønnesby–Borgan test [47]. Internal validation of the model was performed by bootstrapping 100 random samples and subsequent adjustment of the concordance-index [48]. A simplified biomarker-score was developed by allocating points to patients according to individual biomarker measurements.

The additive prognostic capability of our biomarker-based model to an established prognostic model was explored by adjusting analysis for the modified Lung Cancer Prognostic Index (m-LCPI), an externally validated prognostic model in NSCLC based on clinical variables (stage, histology, absence of actionable mutation (EGFR/ALK negative; KRAS positive; mutation not tested), smoking history, respiratory comorbidity, sex, age) [38].

All statistical analyses were performed with the commercially available package STATA 15.0 (Stata Corp., Houston, TX, USA).

## 5. Conclusions

In conclusion, our study strongly supports the concept of an association of haemostatic and inflammatory activation with aggressive clinical behaviour of advanced lung cancer. Further, the strong individual prognostic capability of haemostatic and inflammatory biomarkers can be combined within a biomarker-based prognostic score that efficiently stratifies prognosis beyond patient- and disease-related characteristics in patients with advanced lung cancer.

## Figures and Tables

**Table 1:** Characteristics of patients at study inclusion (*n* = 277).

Variable	*n* (% Missing Values)	Median [IQR] (Range) or Count (%)
Demographics and clinical characteristics		
Age (years)	277 (0%)	61 [56, 67]
Female Gender	277 (0%)	103 (37.2%)
BMI (kg/m^2^)	275 (0.7%)	24.6 [22.2, 28.1]
ECOG	154 (44.4%)	1 [1, 2]
History of smoking	271 (2.1%)	203 (74.9%)
History of VTE *	277 (0%)	9 (3.2%)
m-LCPI for NSCLC **	230 (0.4%)	-
Group 1 (≤8)	-	9 (3.9%)
Group 2 (9–11)	-	36 (15.7%)
Group 3 (12–14)	-	162 (70.4%)
Group 4 (≥15)	-	23 (10.0%)
Tumour specifics at inclusion		
Histology	277 (0%)	-
SCLC	-	46 (16.6%)
NSCLC	-	231 (83.4%)
Adenocarcinoma	-	165 (59.6%)
SCC	-	45 (16.2%)
LCNEC	-	9 (3.2%)
Others	-	12 (4.3%)
Stage	277 (0%)	-
I	-	1 (0.4%)
II	-	4 (1.4%)
III	-	76 (27.4%)
IV	-	196 (70.8%)
Distant metastatic site	-	-
Cerebral	-	51 (18.4%)
Bones	-	63 (22.7%)
Lung contralateral	-	64 (23.1)
Pleural	-	38 (13.7%)
Adrenal	-	36 (13.0%)
Liver	-	24 (8.7%)
Histological grade	196 (29.2%)	-
G1 (well differentiated, low grade)	-	5 (1.8%)
G2 (moderately differentiated, intermediate grade)	-	80 (40.8%)
G3 (poorly differentiated, high grade)	-	101 (51.5%)
G4 (undifferentiated, high grade)	-	10 (5.1%)
Therapeutic management		
Chemotherapy	277 (0%)	277 (100%)
Palliative intent	-	249 (88.8%)
Neoadjuvant intent	-	31 (11.2%)
Surgery (Primary)	277 (0%)	45 (16.2%)
Secondary metastasectomy	-	13 (3.5%)
Radiotherapy	277 (0%)	119 (43.0%)
Cumulative dose	-	60 [30, 90]
Chemotherapy regimen	275 (0.7%)	-
Platin-Vinorelbine	-	74 (26.9%)
Platin-Gemcitabine	-	52 (18.9%)
Platin-Pemetrexed	-	52 (18.9%)
Platin-Etoposid	-	49 (17.8%)
Anti-VEGF-therapy	-	6 (2.2%)
EGFR-TKI	-	10 (3.6%)
Number of chemotherapy cycles	271 (0.7%)	4 [2, 5]
2nd line chemotherapy	-	112 (40.4%)
Checkpoint-inhibitor therapy after chemotherapy	-	6 (2.2%)
Levels of biomarkers prior to initiation of therapy (median [IQR])		
Factor VIII (% activity)	264 (4.7%)	191 [156, 248]
sP-selectin (ng/mL)	275 (0.7%)	42.8 [33.4, 54.4]
D-dimer (µg/mL)	247 (10.8%)	0.88 [0.55, 1.90]
Prothrombin fragment 1+2 (pmol/L)	273 (1.4%)	225 [165, 348]
Fibrinogen (mg/dL)	275 (0.7%)	485 [376, 590]
Peak thrombin generation (nmol/L)	271 (2.2%)	363 [228, 509]
Platelet count (G/L)	275 (0.7%)	294 [241, 352]
Leucocyte count (G/L)	275 (0.7%)	8.34 [6.82–10.37]
Haemoglobin (mg/dl)	275 (0.7%)	12.9 [11.8, 14.0]
Neutrophil granulocytes (G/L)	233 (15.9%)	5.8 [4.5, 7.9]
Lymphocytes (G/L)	232 (16.2%)	1.2 [0.9, 1.6]
Monocytes (G/L)	231 (16.6%)	0.6 [0.4, 0.8]
Neutrophil-to-Lymphocyte Ratio (NLR)	232 (16.2%)	4.8 [3.3, 7.3]
Lymphocyte-to-Monocyte Ratio (LMR)	230 (17.0%)	2.0 [1.4, 3.2]
Platelet-to-Lymphocyte Ratio (PLR)	232 (16.2%)	246.5 [163.3, 337.2]
C-reactive protein (CRP)	250 (9.7%)	1.6 [0.6, 4.5]

* according to CATS, prior VTE and termination of prior chemotherapy had to be ≥3 months before enrolment; ** items included in the m-LCPI: stage, histology (NSCLC not otherwise specified), presence of actionable mutation, history of smoking, respiratory comorbidity, male sex, age group (≤50; 51–70; 71–90; ≥90) Abbreviations: IQR: Interquartile-range; ECOG: Eastern Co-operative of Oncology Group performance index; VTE: venous thromboembolism; m-LCPI: modified lung cancer prognostic index; NSCLC: non-small-cell lung cancer; SCLC: small-cell lung cancer; SCC: squamous-cell carcinoma; LCNEC: large cell neuroendocrine carcinoma; VEGF: vascular endothelial growth factor; EGFR-TKI: epidermal growth factor receptor tyrosine kinase inhibitor.

**Table 2:** Uni- and multivariable association of biomarkers with mortality, progression free survival and disease-control-rate.

Biomarker	HR for Death (Mortality)		HR for Disease Progression (PFS)		OR for Therapy Response (DCR)	
	Uni-Variable	Multi-Variable *	Uni-Variable	Multi-Variable *	Uni-Variable	Multi-Variable *
Haemostatic Biomarkers						
D-dimer	1.58 [1.38–1.81] *p* < 0.001	1.50 [1.29–1.75] *p* < 0.001	1.41 [1.23–1.61] *p* < 0.001	1.34 [1.16–1.53] *p* < 0.001	0.69 [0.50–0.94] *p* = 0.020	0.73 [0.52–1.04] *p* = 0.083
F1+2	1.24 [1.06–1.46] *p* = 0.008	1.15 [0.97–1.36] *p* = 0.098	1.25 [1.07–1.46] *p* = 0.006	1.22 [1.04–1.44] *p* = 0.013	0.69 [0.50–0.97] *p* = 0.031	0.71 [0.50–1.02] *p* = 0.063
sP-selectin	1.42 [1.11–1.83] *p* = 0.006	1.42 [1.09–1.83] *p* = 0.008	1.18 [0.94–1.48] *p* = 0.144	1.03 [0.81–1.30] *p* = 0.785	0.72 [0.47–1.11] *p* = 0.136	0.84 [0.53–1.32] *p* = 0.444
Fibrinogen	1.31 [1.02–1.96] *p* = 0.040	1.38 [0.98–1.93] *p* = 0.064	1.24 [0.94–1.65] *p* = 0.133	1.27 [0.95–1.71] *p* = 0.110	0.56 [0.31–1.00] *p* = 0.050	0.62 [0.33–1.14] *p* = 0.124
FVIII	1.55 [1.17–2.06] *p* = 0.002	1.46 [1.08–1.98] *p* = 0.013	1.22 [0.94–1.57] *p* = 0.131	1.16 [0.88–1.52] *p* = 0.305	0.93 [0.57–1.51] *p* = 0.769	1.02 [0.60–1.72] *p* = 0.954
Peak-TG	1.09 [0.93–1.28] *p* = 0.267	1.06 [0.91–1.24] *p* = 0.467	1.12 [0.97–1.28] *p* = 0.124	1.06 [0.93–1.22] *p* = 0.374	0.82 [0.62–1.08] *p* = 0.160	0.86 [0.65–1.14] *p* = 0.295
Inflammatory and blood count parameter						
Platelet count	1.25 [0.95–1.67] *p* = 0.110	1.28 [0.95–1.73] *p* = 0.102	1.14 [0.89–1.45] *p* = 0.304	1.13 [0.87–1.47] *p* = 0.355	0.66 [0.40–1.11] *p* = 0.116	0.75 [0.45–1.27] *p* = 0.285
Leucocytes	1.17 [0.86–1.59] *p* = 0.311	1.08 [0.79–1.46] *p* = 0.639	1.22 [0.91–1.64] *p* = 0.180	1.11 [0.83–1.49] *p* = 0.496	0.71 [0.41–1.22] *p* = 0.218	0.85 [0.48–1.52] *p* = 0.592
Haemoglobin	0.12 [0.05–0.27] *p* < 0.001	0.13 [0.06–0.30] *p* < 0.001	0.27 [0.13–0.54] *p* < 0.001	0.28 [0.14–0.59] *p* = 0.001	11.32 [2.61–48.97] *p* = 0.001	10.94 [2.26–53.1] *p* = 0.003
Neutrophil granulocytes	1.07 [0.80–1.42] *p* = 0.661	1.05 [0.78–1.41] *p* = 0.760	1.24 [0.95–1.62] *p* = 0.108	1.16 [0.89–1.53] *p* = 0.275	0.74 [0.44–1.24] *p* = 0.257	0.87 [0.50–1.51] *p* = 0.612
Lymphocytes	0.65 [0.52–0.81] *p* < 0.001	0.72 [0.57–0.91] *p* = 0.007	0.65 [0.52–0.79] *p* < 0.001	0.76 [0.61–0.93] *p* = 0.010	1.37 [0.88–2.12] *p* = 0.155	1.18 [0.74–1.88] *p* = 0.486
Monocytes	1.19 [0.99–1.43] *p* = 0.066	1.17 [0.98–1.40] *p* = 0.086	1.05 [0.89–1.24] *p* = 0.553	1.08 [0.93–1.27] *p* = 0.311	1.02 [0.75–1.38] *p* = 0.920	1.06 [0.78–1.46] *p* = 0.700
NLR	1.33 [1.12–1.59] *p* = 0.001	1.24 [1.03–1.49] *p* = 0.024	1.39 [1.19–1.61] *p* < 0.001	1.24 [1.05–1.46] *p* = 0.009	0.74 [0.53–1.03] *p* = 0.071	0.85 [0.60–1.21] *p* = 0.378
LMR	0.52 [0.39–0.69] *p* < 0.001	0.60 [0.45–0.80] *p* = 0.001	0.67 [0.52–0.86] *p* = 0.001	0.74 [0.58–0.94] *p* = 0.013	1.10 [0.72–1.70] *p* = 0.658	0.94 [0.60–1.47] *p* = 0.786
PLR	1.41 [1.17–1.72] *p* < 0.001	1.31 [1.08–1.61] *p* = 0.008	1.38 [1.16–1.65] *p* < 0.001	1.24 [1.03–1.48] *p* = 0.021	0.71 [0.50–1.01] *p* = 0.056	0.80 [0.56–1.16] *p* = 0.239
CRP	1.51 [1.35–1.71] *p* < 0.001	1.45 [1.27–1.65] *p* < 0.001	1.31 [1.18–1.47] *p* < 0.001	1.25 [1.11–1.40] *p* < 0.001	0.64 [0.50–0.81] *p* < 0.001	0.69 [0.53–0.89] *p* = 0.005

* adjusted for stage, histology, presence of druggable mutation, pulmonary comorbidity, age, sex, VTE (as time-dependent covariable in time-to-event analyses); Abbreviations: HR: hazard ratio; PFS: progression free survival; OR: odds ratio; DCR: disease control rate; F1+2: prothrombin fragment 1+2; FVIII: coagulation factor VIII; peak-TG: peak thrombin generation; NLR: neutrophil-to-lymphocyte ratio; LMR: lymphocyte-to-monocyte ratio; PLR: platelet-to-lymphocyte ratio; CRP: C-reactive protein.

## Appendix A

### Biomarker Description and Rationale

D-dimer: A fibrin degradation product, representing systemic coagulation and fibrinolysis activity. D-dimer was associated with survival in a general population of patients with cancer in a prospective study and in lung cancer specifically in retrospective cohort studies [23,26].

Prothrombin fragment 1+2 (F1+2): A by-product of thrombin generation that is cleaved from prothrombin, representing the activity of the prothrombinase complex in vivo. F1+2 was associated with risk of cancer-associated VTE [25].

Coagulation factor VIII activity (FVIII): FVIII acts as a cofactor for the factor IX dependent activation of the prothrombinase complex. Higher levels of FVIII have been observed in patients with cancer and have been linked to the risk of cancer-associated VTE [27].

Peak thrombin generation (peak-TG): The thrombin generation assay represents an in vitro assay, evaluating the potential thrombin generation potential. The peak height of the thrombin generation per time (peak-TG) is a marker for hypercoagulability and has been associated with risk of cancer-associated VTE [28].

Soluble sP-selectin: A marker for platelet and endothelial cell activation in vivo. Levels of sP-selectin have been associated to risk of cancer-associated VTE and prospective risk of developing lung cancer [20,29].

Fibrinogen: The main substrate for coagulation in vivo. Levels of fibrinogen have been associated to the prospective risk of developing lung cancer, survival in patients with lung cancer and with metastasis in experimental models of lung cancer [19,20,24].

C-reactive protein (CRP): An acute-phase plasma protein that is elevated in systemic inflammation and is commonly used as an inflammatory marker. CRP has been associated to primary risk of lung cancer and survival in patients with lung cancer [8,32].

Neutrophil-to-lymphocyte ratio (NLR): One of the cell-count based markers representing systemic inflammation. NLR has been associated with poor prognosis in various cancers, including lung cancer [11].

Lymphocyte-to-monocyte ratio (LMR): One of the cell-count based markers representing systemic inflammation. LMR has been demonstrated to be prognostic in numerous cancers, including advanced lung cancer [17].

Platelet-to-lymphocyte ratio (PLR): One of the cell-count based markers indicating systemic inflammation. PLR was associated with survival in patients with lung cancer [12].

Haemoglobin: Systemic inflammation and malignancy can induce anaemia among others by cytokine-induced impairment of erythropoiesis, increased decay of erythrocytes and hepcidin-induced hypoferremia. Due to the in- and exclusion criteria of our study, iatrogenic factors that might alter systemic levels of markers including haemoglobin (e.g., chemotherapy within 3 months prior to inclusion) are avoided. Therefore, decrease in haemoglobin can be regarded as an indicator of chronic disease and chronic inflammation [33]. Decreased levels of pre-treatment haemoglobin were associated with survival in patients with lung cancer [34].

Leucocyte count: An indicator of systemic inflammatory activation that has been associated with prognosis in various cancers and oncologic settings including lung cancer [35].

Thrombocyte count: Platelets are involved in cancer cell proliferation, tumour progression and the metastatic process. Thrombocytosis has been linked to worse survival in patients with lung cancer [36].

## Appendix B

### Vienna Cancer and Thrombosis Study (CATS): Design, in-/Exclusion

Design: single centre, prospective, observational cohort study

Study centre: Medical University of Vienna, Vienna, Austria

Ethics committee and vote: ethics committee of the Medical University of Vienna (number: 126/2003, ethik–kom@meduniwien.ac.at); conducted in accordance with the declaration of Helsinki

Study timeframe: start: October 2003, enrolment still ongoing

Primary aim of the study: Identification of predictive parameters for the occurrence of VTE in patients with cancer

Inclusion criteria:

- patients with newly diagnosed cancer of the brain, breast, lung, upper or lower gastrointestinal tract, pancreas, kidney, prostate or gynaecologic system; sarcoma; hematologic malignancies (myeloma, high- and low-grade lymphoma); or progression of disease after complete or partial remission;
- histologic confirmation of diagnosis;
- age more than 18 years;
- willingness to participate;
- written informed consent.

Exclusion criteria

- overt bacterial or viral infection within the last 2 weeks;
- venous or arterial thromboembolism within the last 3 months;
- continuous anticoagulation with vitamin K antagonists or low molecular weight heparin (LMWH); patients were allowed to take aspirin, ticlopidine, or clopidogrel, and immobilized patients were treated with LMWH as thrombosis prophylaxis during their hospital stay;
- surgery or radiotherapy within the last 2 weeks;
- chemotherapy within the last 3 months.

For inclusion in the study, all inclusion criteria had to be fulfilled; one exclusion criterion sufficed for exclusion.

## Appendix C

### Biomarker Measurement: Methods and Timepoints

Blood sampling was conducted at baseline, prior to the initiation of systemic anticancer therapy. Biomarker analysis was conducted either the same day or in series from stored aliquots. Haemostatic biomarkers were measures from venous blood samples collected into Vacutainer citrate tubes (Vacuette; Greiner-Bio-One; containing 1/10 volume sodium citrate stock solution at 0.129 mmol/L) by sterile, atraumatic venipuncture. After centrifugation at 3000× *g* for 10 min to obtain platelet-poor plasma testing was performed, or aliquots were stored at −80 °C. Testing at the day of inclusion was conducted for FVIII and fibrinogen. Testing was performed from stored aliquots in series for D-dimer, F1+2 and peak-TG. To obtain platelet-free plasma, a second centrifugation step (Epppendorf) at 13,400× *g* for 2 min was performed. Platelet-free plasma aliquots were stored until measurement of sP-selectin was performed in series. The following methods were used for individual biomarker measurements:

- D-dimer: Quantitative latex assay (STA-LIAtest D-DI; Diagnostica-Stago, Asnieres, France) on an STA-R analyser (Diagnostica-Stago).
- F1+2: Enzyme-linked immunoassay (Enzygnost F1+2; Dade-Behring, Marburg, Germany).
- FVIII: Sysmex CA 7000 analyser using factor VIII-deficient plasma (Technoclone, Vienna, Austria) and APTT Actin-FS (Dade-Behring).
- Peak-TG: Technothrombin TGA kit, (Technoclone) on a fully automated, computer-controlled microplate reader (BioTek, FL ×800, Winooski, Vermont, USA) and a specially adapted software (Technothrombin TGA, Vienna, Austria) using the fluorogenic substrate Z-Gly-Gly-Arg-AMC (Bachem, Bubendorf, Switzerland); The reaction was triggered with the TGA RC low reagent, which contained 71.6 pM recombinant human tissue factor lipidated in 3.2 μmol/L phospholipid micelles (phosphatidylcholine [2.56 μmol/L] and phosphatidylserine [0.64 μmol/L]).
- sP-selectin: human sP-selectin Immunoassay (R&D Systems, Minneapolis, MN, USA)
- Fibrinogen: routinely measured in platelet poor plasma according to Clauss (STA Fibrinogen; Diagnostica-Stago).

### Inflammatory Biomarkers and Blood Count Parameters:

Inflammatory markers and blood count parameters were measured on the day of study inclusion by routine testing of blood samples at the Department for Laboratory Medicine of the Medical University of Vienna. CRP was measures from blood collected in Vacutainer serum tubes and haemoglobin, leucocytes, thrombocytes and leucocyte subpopulations was measured from venous blood collected in Vacutainer EDTA-tubes. The following methods were used for individual biomarker measurements:

- CRP: Immunoturbidimetry.
- Blood count parameters (leucocyte count, leucocyte subpopulations, thrombocyte count, haemoglobin): Sysmex XE-5000/XN-1000/XN-2000.
- NLR: Calculated ratio of absolute neutrophil count to absolute lymphocyte count.
- LMR: Calculated ratio of absolute lymphocyte count to absolute monocyte count.
- PLR: Calculated ratio of absolute platelet count to absolute lymphocyte count.
